# Methylation analysis of HOXA10 regulatory elements in patients with endometriosis

**DOI:** 10.1186/s13104-018-3836-1

**Published:** 2018-10-11

**Authors:** Pietro G. Signorile, Anna Severino, Massimo Santoro, Maria Spyrou, Rosa Viceconte, Alfonso Baldi

**Affiliations:** 1 Fondazione Italiana Endometriosi, Rome, Italy; 2 Fondazione Policlinico Universitario A. Gemelli-IRCCS, Rome, Italy; 3 IRCCS Fondazione Don Carlo Gnocchi, Milan, Italy; 4 Department of Environmental, Biological and Pharmaceutical Sciences and Technologies, University of Campania “L. Vanvitelli”, Caserta, Italy

**Keywords:** Endometriosis, HOXA10 gene promoter, DNA methylation analysis, CpG islands

## Abstract

**Objective:**

The pathogenesis of endometriosis is still mysterious, being retrograde menstruation and coelomic metaplasia the most accepted hypotheses. Recently, it has been proposed that endometriosis is caused by fine-tuning alterations of the female genital system development during the foetal life and that in utero exposition to endocrine disruptors can be one of the factors causing the disease, possibly acting on the methylation status of the genome. In this study, we have evaluated the methylation status of *HOXA10* gene regulation regions in a cohort of 22 endometriosis patients respect to a control group of 6 healthy women.

**Results:**

The methylation study was carried out on three CpG islands, previously described hypermethylated in the endometrium of endometriosis patients and include 22 CpG sites, 21 CpG sites and 10 CpG sites, respectively identified through the online platform MethPrimer. The analysis did not find significant differences between patients with endometriosis and healthy control individuals. These results confirm previous studies on genome wide methylation analysis in endometriosis patients. Therefore, other epigenetically altered genes should be considered more related to the pathogenesis of endometriosis.

## Introduction

Endometriosis is a pathological condition characterized by the the presence of endometrial glands and stroma outside of the uterine cavity. It is a gynaecological disease, that affects a significant number of reproductive-aged women [1, 2]. Endometrial implants can cause substantial morbidity, and infertility, often necessitating extensive medical and surgical treatments with significant costs and risks [3]. The pathogenesis of the disease is still mysterious. Retrograde menstruation and coelomic metaplasia are, actually, the most accepted hypotheses [4]. Recently, our research group has produced evidences underlining the possibility that endometriosis is caused by fine-tuning alterations of the female genital system development during the foetal life [5–9]. In detail, the commencement and evolution of this phenomenon may result from disruptions by genetic and/or epigenetic factors of some organizational events associated with the development of the embryonic uterine wall. This theory is also supported by a recent work in which we have described an endometriosis-like phenotype in mice exposed in utero to the endocrine disruptor bisphenol (BPA) [10]. Indeed, it is well demonstrated the fact that pre- and/or peri-natal exposure to BPA has effects on the genital system that persists in the adult [11]. The molecular mechanisms responsible of this phenomenon include alterations of HOX gene expression in the developing mullerian system [12]. HOX genes play a fundamental role in the correct axial development of the primitive Mullerian duct in the fallopian tubes, uterus, cervix and upper vagina [13]. They are highly evolutionarily conserved transcription factors and are expressed in a temporally and spatially linear manner [12]. Aberrant DNA methylation is a molecular mechanism, possibly connecting gene expression alterations observed in endometriosis with hormonal and environmental factors [14]. Interestingly, it has been shown that in endometriosis patients a decrease in the expression of HOXA10 has been detected in the endometrium during the secretory phase [15]. Hypermethylation of the HOXA10 gene promoter is, indeed, one of the potential molecular mechanisms silencing HOXA10 expression in the endometrium of patients with endometriosis [16]. Supporting this hypothesis there are several studies showing HOXA10 promoter hypermethylation and reduced HOXA10 expression in experimental endometriosis in baboons and in mice [17, 18]. Nevertheless, mice, prenatally exposed to the endocrine disruptor diethylstilbestrol, displayed HOXA10 hypermethylation [19]. Leaving from this background, in this work, we have analyzed the methylation status of *HOXA10* gene regulation regions in a cohort of endometriosis patients respect to a control group of healthy women.

## Main text

Blood samples were collected at the “Centro Italiano Endometriosi” in Rome from volunteer healthy controls and from women affected by endometriosis that underwent surgery for adnexal masses, infertility, pelvic pain symptoms (including dysmenorrhea, deep dyspareunia and no-menstrual pain). The diagnosis of endometriosis was confirmed by histological analysis in all the selected patients. Genomic DNA was extracted from 6 ml of peripheral anti-coagulated blood (EDTA) from patients (n = 20) and healthy controls (n = 6) using the blood DNA extraction kit (Qiagen, Germany) following the manufacturer’s instructions. The genomic DNA was quantified by the Qubit^®^ 2.0 Fluorometer (ThermoFisher Scientific, USA) according to the manufacturer’s instructions.

**Fig. 1:**
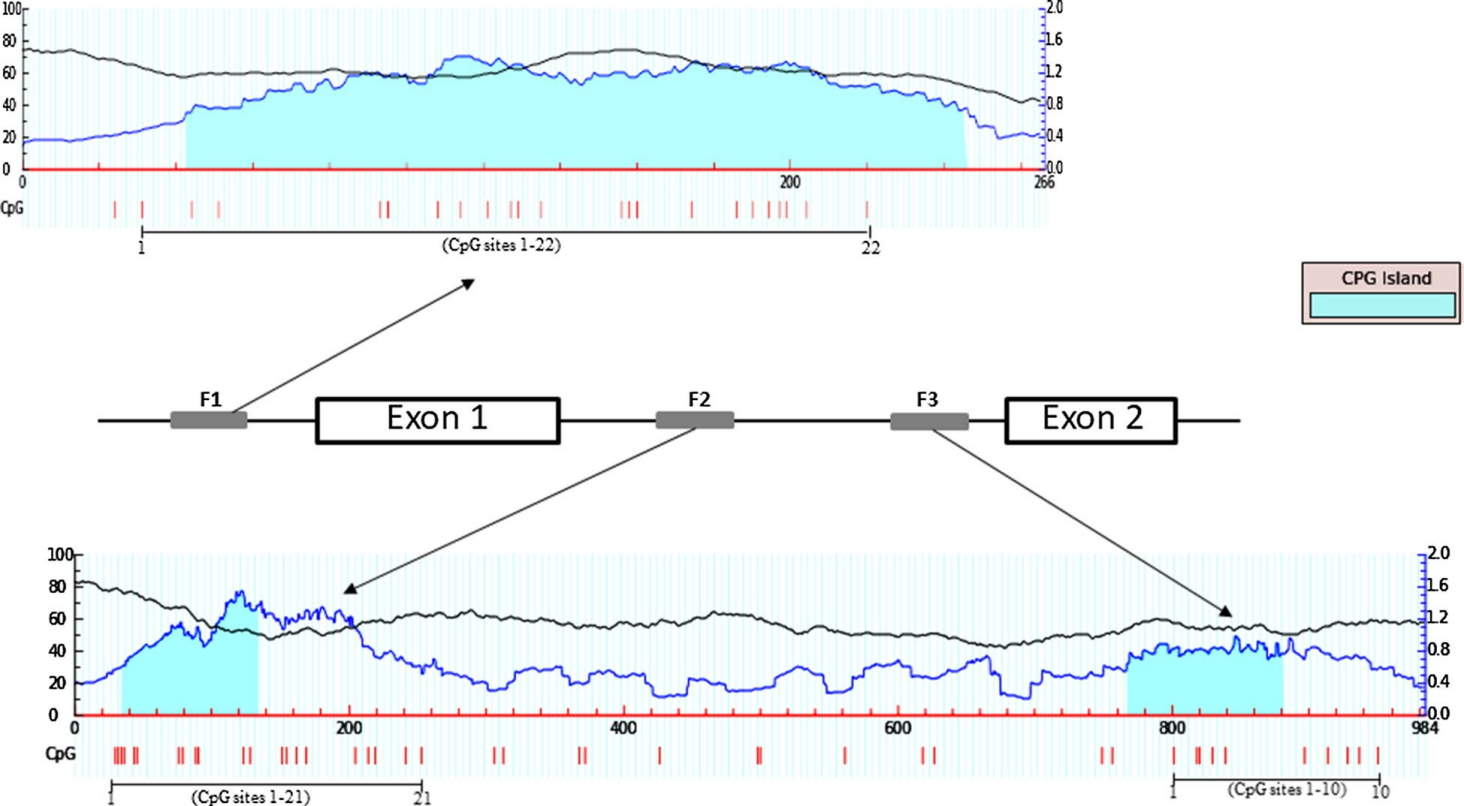
Genomic organization of *HOXA10* locus used for methylation analysis. CpG island (blue region) identified by MethPrimer program (upper and lower panel). Vertical red bars indicate relative positions of CpG sites that are numbered from 1 to 22 for fragment F1 (gray box), from 1 to 21 for fragment F2 (gray box) and from 1 to 10 for fragment F3 (gray box). Exon sequences are indicated as white box and intronic sequences as thick black line

The methylation status of a DNA sequence can best be determined using sodium bisulfite. Incubation of the target DNA with sodium bisulfite results in conversion of unmethylated cytosine residues into thymines, leaving the methylated cytosines unchanged. Therefore, bisulfite treatment gives rise to different DNA sequences for methylated and unmethylated DNA. 2 µg of gDNA were used for bisulfite conversion by the Epitect 96 Bisulfite kit (Qiagen) following the manufacturer’s instruction. After bisulphite conversion, the genomic DNA was quantified by the Qubit^®^2.0 Fluorometer (ThermoFisher Scientific, USA). The methylation study was carried out on three CpG islands previously described [20] and identified through the online platform MethPrimer (http://www.urogene.org/methprimer/. The first CpG island (indicated as fragment F1) was located within 5′ upstream region of *HOXA10* exon1 gene (accession number: AF040714, position 23–289) including 22 CpG sites (Fig. 1 upper panel). The second and the third CpG islands (indicated as fragments F2 and F3 respectively) were located in the intron region of *HOXA10* gene and containing 21 and 10 CpG sites respectively (accession number: AF040714, positions 1520–1811 and 2293–2509 respectively) (Fig. 1 lower panel). These three CpG islands have been previously described hypermethylated in the endometrium of endometriosis patients [21]. Fragments of 266 bp (5′ upstream region), of 291 bp (intron region) and of 216 bp (intron region) were amplified by PCR from bisulphite-treated DNA (60 ng) using 3 set of primers previously described [20]. EpiTect PCR Control DNA Set (Qiagen, Germany), containing both bisulphite converted methylated and unmethylated DNA was included in experimental design as technical control. The methylation status was assessed by bisulfite sequencing, which is considered to be the gold standard for methylation evaluation. PCR amplified fragments were purified with PCR QIAquick PCR purification kit (Qiagen) following the manufacturer’s instructions and sequenced by Eurofins MWG operon service (M-Medical).

Methylation percentage of single CpG sites was calculated considering chromatogram peaks for thymine (T) (representing unmethylated cytosines) and cytosine (C) (representing methylated cytosines). Cytosine methylation (%) = [C/(C + T)] × 100 [21]. The methylation analysis was performed using both forward and reverse primers [22]. By sequencing analysis, we found in the fragment F1 an average methylation level of 4–5% in both controls and endometriosis patients (Fig. 1; Table 1). On the other hand, all CpG sites within fragments F2 and F3 were hypomethylated with no significant differences between patients with endometriosis and in healthy control individuals (Fig. 1; Tables 1, 2). Both bisulfite converted methylated and unmethylated DNA was included in experimental design as technical control.

**Table 1:** Methylation percentage average of single CpG sites at 5′ upstream region of *HOXA10* exon1 (fragment F1) and of single CpG sites at intron region of *HOXA10* exon1 (fragment F2) in leucocytes from controls (CTRL) and endometriosis patients (Pts)

	CTRL (*n *= 6) Fragment 1	Pts (*n *= 22) Fragment 1	CTRL (*n *= 6) Fragment 2	Pts (*n *= 22) Fragment 2
CpG1	4	1	4	3
CpG2	3	7	2	2
CpG3	6	4	4	1
CpG4	8	8	5	2
CpG5	3	4	3	2
CpG6	2	4	1	2
CpG7	6	6	7	3
CpG8	8	8	5	3
CpG9	10	9	4	2
CpG10	3	3	3	4
CpG11	5	6	2	4
CpG12	6	5	2	3
CpG13	2	1	1	3
CpG14	5	3	4	2
CpG15	2	6	1	1
CpG16	2	3	2	2
CpG17	6	7	4	1
CpG18	7	6	5	1
CpG19	4	5	3	4
CpG20	5	4	2	4
CpG21	2	6	1	3
CpG22	1	5	1	3
CpG average	4.5	5	3.1	2.5

**Table 2:** Methylation percentage average of single CpG sites at intron region of *HOXA10* exon1 (fragment F3) in leucocytes from controls (CTRL) and endometriosis patients (Pts)

	CTRL (*n *= 6)	Pts (*n *= 22)
CpG1	2	2
CpG2	3	2
CpG3	3	2
CpG4	3	3
CpG5	2	3
CpG6	2	1
CpG7	3	3
CpG8	4	2
CpG9	5	4
CpG10	2	2
CpG average	2.9	2.4

Several evidences suggest a multifactorial origin of endometriosis; consequently, a linear association between environmental factors, epigenetic alterations and disease, is difficult to define. Nevertheless, endometriosis has been often linked to exposure to toxins or synthetic compounds and dietary habits [10, 11, 14], and DNA methylation has been proposed as one of the molecular mechanisms causing selective epigenetic deregulations influenced by extrinsic factors [23]. Recent theories on fetal programming suggest that chronic adult onset diseases with an epigenetic component, initiate in fetal life with the exposition of the early embryo to factors that permanently shape its epigenetic mark [24, 25]. The demonstration of an endometriosis phenotype in fetuses as a well as of an endometriosis-like phenotype in mice exposed in utero to BPA, are coherent with this theory [11]. Combining this observation with the fact that in patients with endometriosis there is a decrease in HOXA10 expression, at least in part responsible of the insufficient uterine receptivity of endometriosis patients [16], makes HOXA10 a potential target of aberrant methylation in endometriosis. In line with the hypothesis of a pathogenetic mechanism for endometriosis that occurs at an embryonic level, in this study we preferred to analyze the methylation status of the HOXA10 gene in the peripheral blood and not in the endometrial tissue. In fact, we were interested not in epigenetic phenomena related to specific tissues, but in molecular mechanisms involving the whole organism in very early stages of embryogenesis. To note, in this study we did not find significant differences in methylation status between patients with endometriosis and healthy control individuals. This observation confirms a recent genome‑wide methylation study in endometriosis patients, where the epigenetic alteration of the HOXA10 gene was below the threshold set for the analysis [26]. Therefore, even though hypermethylation of the HOXA10 promoter has been frequently found in different studies on endometriosis, other epigenetically altered genes should be considered more related to the pathogenesis of this disease. In conclusion, although several studies, utilizing cutting-edge molecular techniques, have consistently shown a possible association of DNA methylation with altered gene expression in endometriosis [27], whether this phenomenon represents the cause or the consequence of the disease is a question which remains to be responded.

## Limitations

Major limitation of this study is the limited number of endometriosis patients enrolled in the study. This does not allow to reach definitive conclusions. However, as discussed in advance, the data presented are in accordance with recent genome‑wide methylation study of the HOXA10 gene in endometriosis patients.

## Abbreviation

BPA: bisphenol
